# A Highly Sensitive and Selective Competition Assay for the Detection of Cysteine Using Mercury-Specific DNA, Hg^2+^ and Sybr Green I

**DOI:** 10.3390/s111110187

**Published:** 2011-10-26

**Authors:** Hui Xu, Shuli Gao, Quanwen Liu, Dun Pan, Lihua Wang, Shuzhen Ren, Min Ding, Jingwen Chen, Gang Liu

**Affiliations:** 1 School of Chemistry and Material Sciences, Ludong University, Yantai 264025, China; E-Mails: shuli0049@126.com (S.G.); ldu_lqw@163.com (Q.L.); 2 Laboratory of Physical Biology, Shanghai Institute of Applied Physics, Chinese Academy of Sciences, Shanghai 201800, China; E-Mails: pandun@sinap.ac.cn (D.P.); Wanglihua@sinap.ac.cn (L.W.); 3 Shanghai Institute of Measurement and Testing Technology, Shanghai 201203, China; E-Mails: renshuzhen@simt.com.cn (S.R.); dingmin@simt.com.cn (M.D.); chendingwen@simt.com.cn (J.C.)

**Keywords:** cysteine, competition assay, mercury-specific DNA, Hg^2+^, Sybr Green I

## Abstract

We here report a rapid, sensitive, selective and label-free fluorescence detection method for cysteine (Cys). The conformation of mercury-specific DNA (MSD) changes from a random coil form to a hairpin structure in the presence of Hg^2+^ due to the formation of a thymine-Hg^2+^-thymine (T-Hg^2+^-T) complex. Cys can selectively coordinate with Hg^2+^ and extract it from the thymine-Hg^2+^-thymine complex. The hairpin structure dehybridizes and the fluorescence intensity of Sybr Green I (SG) decreases upon addition of Cys because SG efficiently discriminates mercury-specific DNA and mercury-specific DNA/Hg^2+^ complex. The detection can be finished within 5 min with high sensitivity and selectivity. In addition, we can obtain variable dynamic ranges for Cys by changing the concentration of MSD/Hg^2+^.

## 1. Introduction

Widely distributed in living cells, thiols such as cysteine (Cys) are involved in many biological functions. Their levels in biological fluids such as human plasma and urine are of great importance for clinical diagnostics of a variety of diseases [1–3]. Determination of these species can be achieved by effective separation/detection techniques, e.g., HPLC and capillary electrophoresis [4–6], spectrophotometry [7–9], electrochemical voltammetry [10–13], colorimetric methods [14–17], flow injection [18–20] and fluorescence analysis method [21–25]. However, some of them suffer from low sensitivity and/or selectivity or require cumbersome laboratory procedures. There is thus an intense demand for more sensitive, selective, convenient and low-cost methods to detect Cys.

Growing research interest has been focused on the development of sensitive, selective, and cost-effective biosensors based on target-responsive DNA structural switching [26–29]. The core technology of these sensors is a kind of special DNA molecules which change their conformations upon binding with the targets. For example, aptamers are *in vitro* selected functional oligonucleotides that can bind specifically to target molecules. In this work, we use a mercury-specific DNA (MSD) which presents a random coil form in the absence of Hg^2+^, and forms a hairpin structure in the presence of Hg^2+^ due to the formation of a thymine-Hg^2+^-thymine (T-Hg^2+^-T) complex. A fluorescent dye, Sybr Green I (SG), was applied to recognize the structural change due to the different interaction of SG with MSD and MSD/Hg^2+^ complex [30].

It is well-known that Cys can form a very stable complex with Hg^2+^ [31]. By using this property, Mirkin *et al.* [32] developed a highly sensitive and selective colorimetric detection method for Cys. However, their method needs an elevated temperature, which requires a long time. We recently developed a fluorescent turn-on “molecular beacon” probe for the detection of Cys, which is also based on the competitive ligation of Hg^2+^ ions by Cys and thymine-thymine (T-T) mismatches [33]. The method shows high sensitivity and selectivity, but still needs to be improved in terms of cost and convenience due to its requirement of a labelled “molecular beacon” and a solution heating process. Here we develop a simple, rapid, sensitive and selective method for detection of Cys by using a target-responsive DNA structural change. The MSD/Hg^2+^ complex is a hairpin structure, which dehybridizes when Hg^2+^ is extracted from the thymine-Hg^2+^-thymine complex by Cys due to the high formation constant of Hg^2+^-Cys complexes. The fluorescence intensity of SG then decreases upon addition of Cys due to the dehybridization of the MSD/Hg^2+^ complex.

## 2. Experimental Section

### 2.1. Chemicals and Apparatus

All chemicals used for these investigations were of analytical grade purity. L-Cysteine (Cys, minimum 98.5%) was purchased from Sigma Aldrich Chemical Company and used as received. MSD (5′-TTCTTTCTTCCCCTTGTTTGTT-3′) was synthesized and purified by HPLC (Takara Biotech. Co., Dalian). SG (10,000×) was purchased from Invitrogen Inc. A stock solution of 400× was prepared with DMSO/water (volume 1:1) before use. Milli-Q water (18.2 MΩ cm) was used in all procedures. The fluorescence measurements were recorded at room temperature (RT) on a Perkin Elmer LS-55 spectrophotometer equipped with a xenon lamp excitation source. Fluorescence spectra were measured at an excitation wavelength of 490 nm and the emission range from 500 to 650 nm with the excitation and emission slit widths set at 5 nm.

### 2.2. Performance of Cys Detection

10 nM MSD was first incubated with 70 nM Hg^2+^ solution in 2.0 mL of 10 mM MOPS (3-(N-morpholino)propanesulfonic acid) buffer containing 0.1 M NaNO_3_ (pH 7.50). 5 μL of 25 × SG was then added to the solution. After incubation for two minutes, different amounts of Cys were added to the solution. The mixture was then immediately used for the fluorescence measurements. For selectivity analysis, various kinds of amino acids with final concentration of 140 nM were used instead of Cys.

## 3. Results and Discussion

### 3.1. Sensor Operation Principle

**Scheme 1.**
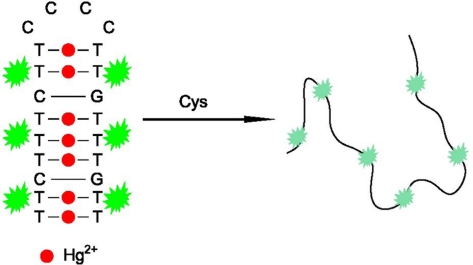
Schematic illustration of the fluorescent Cys detection.

MSD, including seven T-T mismatches, could form a stable hairpin structure upon combination with Hg^2+^, which could be specifically stained by SG and produce a high fluorescence signal. However, in the presence of Cys, Hg^2+^ was extracted from the T-Hg^2+^-T structure because Cys can bind to Hg^2+^ with higher affinity (the formation constant for Cys to Hg^2+^ is *ca.* 10^42^) [31]. As a result, the hairpin structure dehybridizes and the fluorescence intensity of SG decreases. Scheme 1 depicts the designed fluorescence method for Cys detection. The detection can be completed in less than 5 min.

**Figure 1.**
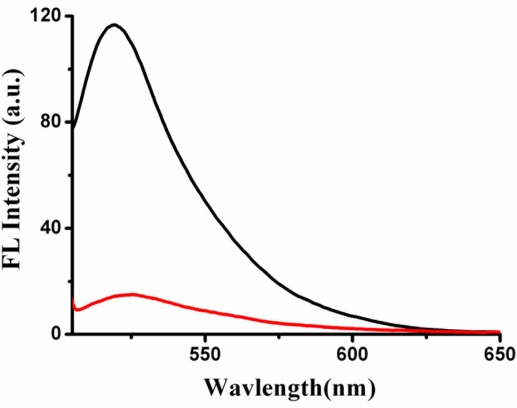
Fluorescence spectra of SG-Hg^2+^-MSD in the absence (black line) and in the presence of Cys (red line). [MSD] = 10 nM, [Hg^2+^] = 70 nM, [SG] = 1.225 × 10^−7^ M, [Cys] = 140 nM.

As shown in Figure 1, the fluorescence intensity of MSD/Hg^2+^/SG is 116.7 a.u., while MSD/Hg^2+^/SG/Cys is about 15 a.u. when the concentration of Cys is 140 nM.

### 3.2. Optimization of the Assay

**Figure 2.**
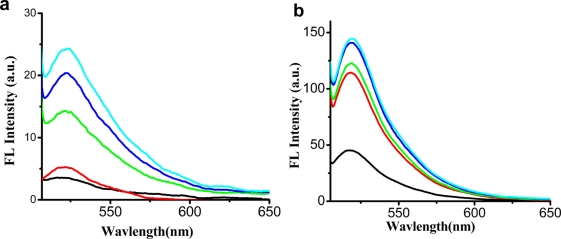

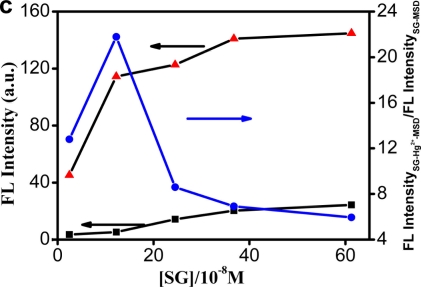
Fluorescence spectra of MSD **(a)** and MSD-Hg^2+^ **(b)** upon addition of different concentration of SG. [MSD] = 10 nM, [Hg^2+^] = 70 nM, [SG](from bottom to up) = 2.45, 12.25, 24.5, 36.75, 61.25 × 10^−8^ M, respectively. **(c)** Relationship between fluorescence intensity and the concentration of SG for SG-Hg^2+^-MSD and SG-MSD. Squares and triangles represent the fluorescence intensity of SG-MSD and SG-Hg^2+^-MSD, respectively; circles are the ratios of the fluorescence intensity SG-Hg^2+^-MSD to that of SG-MSD. 10 mM MOPS, 0.1 M NaNO_3_, pH 7.50 was used as the buffer.

In previous study, Liu *et al.* [30] found that MSD kinetically accomplishes the hairpin structure at [Hg^2+^]/7[MSD] = 1. One equivalent of MSD thus requires seven equivalents of Hg^2+^ to completely form the hairpin structure due to the T-Hg^2+^-T chemistry. So we fixed the concentration of MSD to be 10 nM and Hg^2+^ 70 nM, and then the concentration of SG was optimized. Figure 2 shows the fluorescence spectra of MSD and MSD-Hg^2+^ upon addition of different concentration of SG.

We can see from the results that the fluorescence intensity of SG increases both in the absence of Hg^2+^ [Figure 2(a)] and in the presence of Hg^2+^ [Figure 2(b)]. The highest ratio of the fluorescence intensity of SG-Hg^2+^-MSD to that of SG-MSD is 21.8 [Figure 2(c)], at which the concentration of SG is 1.225 × 10^−7^ M, so we selected the concentration of SG to be 1.225 × 10^−7^ M when the concentration of MSD is 10 nM and Hg^2+^ 70 nM.

### 3.3. Cys Sensor Sensitivity

**Figure 3.**
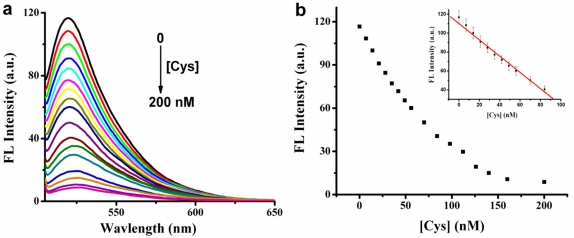
**(a)** Fluorescence spectra of SG-MSD-Hg^2+^ in the absence and presence of Cys, the concentration of Cys (from top to bottom): 0, 7, 14, 21, 28, 35, 42, 49, 56, 70, 84, 98, 112, 126, 140, 160, 200 nM. **(b)** The fluorescence intensity of SG-MSD-Hg^2+^ *vs.* [Cys]. [MSD] = 10 nM, [Hg^2+^] = 70 nM, [SG] = 1.225 × 10^−7^ M. (b inset: the linear relationship of the fluorescence intensity of SG *vs.* [Cys], The error bars represent the standard deviation of three measurements).

To evaluate the sensitivity of the assay, different concentrations of Cys were mixed with MSD/Hg^2+^/SG, and then the fluorescence intensity was immediately measured. As shown in Figure 3(a), the fluorescence intensity of SG decreases gradually upon adding increased concentrations of Cys.

When the concentration of Cys is two-fold higher than that of Hg^2+^, the fluorescence intensity decreases very slowly due to formation of a 2:1 Cys/Hg^2+^ adduct [31]. This implies that almost all the Hg^2+^ has been extracted from T-Hg^2+^-T complex when the concentration of Cys is two fold higher than that of Hg^2+^. When we increased the concentration of Cys further, we found that the fluorescence intensity decreased very slowly. Perhaps some Cys forms Hg(Cys)_3_ complexes. By measuring the fluorescence intensity at the emission maximum of SG-Hg^2+^-MSD-Cys, a linear response of fluorescence intensity *vs.* [Cys] was observed in the range 7–84 nM [Figure 3(b) inset]. The detection limit may be estimated from Equation (1):

(1) $$\text{LOD}=3\times \frac{S_{0}}{S}$$

where S_0_ is the standard deviation of the blank and S is the sensitivity. 3.39 nM was the experimentally estimated detection limit for Cys, which was lower than most reported Cys sensors. Table 1 lists a comparison of methods for the determination of Cys and confirms the results.

What is more important, our method is really fast. In the first step of this assay, SG staining was finished in 2 min, which was proved to be fully adequate for the SG binding by a previous study [30]. In the second step, after the addition of Cys, the fluorescence spectra was measured immediately, and as anticipated, the fluorescence intensity remained stable for a long time (data not shown), because Cys can bind to Hg^2+^ very strongly [31].

**Figure 4.**
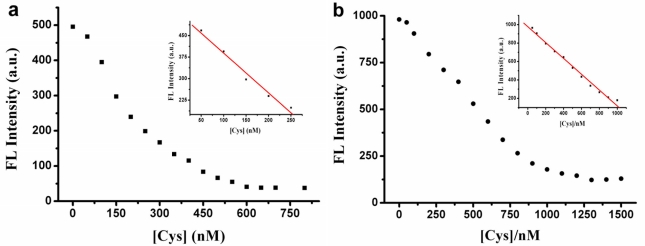
The fluorescence intensity of SG-MSD-Hg^2+^ *vs.* [Cys] **(a)** [MSD] = 50 nM, [Hg^2+^] = 350 nM, [SG] = 6.125 × 10^−7^ M, **(b)** [Cys] = 100 nM, [Hg^2+^] = 700 nM, [SG] = 1.225 × 10^−6^ M (a and b inset: the linear relationship of the fluorescence intensity of SG *vs.* [Cys]).

We further find that we can obtain different dynamic ranges by changing the concentration of MSD/Hg^2+^. For example, when we fixed the concentration of MSD to be 50 nM and that of Hg^2+^ to be 350 nM (MSD kinetically forms the hairpin structure at [Hg^2+^]/7[MSD] = 1 [30]), a linear dynamic range for Cys from 50 to 250 nM was obtained [Figure 4(a) inset]. When we changed the concentration of MSD and Hg^2+^ to be 100 nM and 700 nM, a very wide linear dynamic range for Cys from 50 to 1,000 nM was obtained [Figure 4(b) inset]. The concentration of SG was 6.125 × 10^−7^ M [Figure 4(a)] and 1.225 × 10^−6^ M [Figure 4(b)], respectively, which was determined by the same method mentioned above (Section 3.2). When the concentration of MSD/Hg^2+^ changed, different concentration of Cys was needed to extract Hg^2+^ from T-Hg^2+^-T structure due to the 2:1 Cys/Hg^2+^ adduct, which leads to the different dynamic ranges for Cys.

### 3.4. Cys Sensor Selectivity

**Figure 5.**
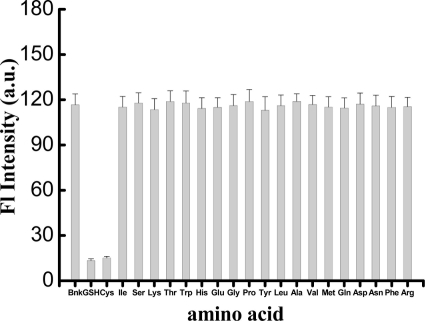
The fluorescence intensity of the blank (SG-MSD-Hg^2+^, no amino acids) and solutions containing different amino acids (SG-MSD-Hg^2+^-amino acid). [MSD] = 10 nM, [Hg^2+^] = 70 nM, [SG] = 1.225 × 10^−7^ M, [amino acids] = [GSH] = 140 nM.

To challenge the assay’s selectivity, other amino acids and the Cys-containing tripeptide glutathione (GSH) at a concentration of 140 nM were analyzed. The results are shown in Figure 5. It is clear that only Cys and GSH show significant fluorescence intensity changes. In contrast to the enormous fluorescence decrease observed for Cys and GSH, there is very little fluorescence change observed in the presence of other amino acids. A Cys-containing tripeptide GSH (γ-Glu-Cys-Gly) showed almost the same fluorescence decrease as Cys, indicating that Hg^2+^ can be extracted from the T-Hg^2+^-T structure upon adding GSH. This is because GSH can also form a very stable 2:1 GSH/Hg^2+^ complex with Hg^2+^ [34–36]. Hg^2+^ is well known to have an affinity to certain N-type ligands such as amino acids [37], but the amino acids, except for Cys, cannot extract Hg^2+^ from the thymine-Hg^2+^-thymine complex. The binding affinity of Hg^2+^ to T-T mismatch sites thus appears to be stronger enough than that of Hg^2+^ to all of the amino acids studied, except Cys. We also found from Figure 5 that a sulfur-containing amino acid like methionine did not result in a significant fluorescence decrease. The formation constant for Cys to Hg^2+^ (about 10^42^) is much higher than that of methionine to Hg^2+^ (about 10^17.6^) [31]. The method presented here therefore shows very high specificity to Cys.

## 4. Conclusions

This paper describes a rapid, highly selective and sensitive fluorescence assay for Cys using mercury-specific DNA and SG. This assay is based on the extraction of Hg^2+^ by Cys from a T-Hg^2+^-T complex. The whole procedure can be done within 5 min. In addition, the assay is label free, low-cost and can provide variable linear dynamic ranges.

## Figures and Tables

**Table 1.** Comparison of methods for the determination of Cys.

**Detection method**	**Linear range (μmol L^−1^)**	**Detection limit (μmol L^−1^)**	**Reference**
Spectrophotometry	0.0082–0.12	0.0049	[9]
Spectrophotometry	0.17–50	Not given	[7]
Fluorimetry	100–5,000	Not given	[21]
Fluorimetry	0.05–4	0.025	[22]
Fluorimetry	0.025–6	0.02	[23]
Fluorimetry	0.01–0.8	0.0038	[24]
Fluorimetry	0.05–0.3	0.0096	[25]
Flow injection	0.001–0.5	0.0005	[18]
Flow injection	1–90	0.2	[19]
Flow injection	0.4–40	0.1	[20]
Capillary zone electrophoresis	0.1–100	0.058	[5]
Voltammetry	0.5–100	0.2	[12]
Voltammetry	2–10,000	1.0	[13]
Voltammetry	0.78–200	0.26 ± 0.01	[10]
Voltammetry	0.5–630	0.07	[11]
Fluorimetry	0.007–0.084 or 0.05–0.25 or 0.05–1	0.0034	This method
